# Engineered soluble truncated envelope proteins block bovine leukemia virus infection

**DOI:** 10.1016/j.virusres.2026.199701

**Published:** 2026-02-05

**Authors:** Nashon Wanjala, Ryusuke Matsumoto, Didik Pramono, Ariko Miyake, Kazuo Nishigaki

**Affiliations:** a Laboratory of Molecular Immunology and Infectious Disease, Joint Graduate School of Veterinary Medicine, Yamaguchi University, 1677-1 Yoshida, Yamaguchi 753-8515, Japan; b Research Institute for Cell Design Medical Science, Yamaguchi University, 1677-1 Yoshida, Yamaguchi 753-8515, Japan

**Keywords:** Bovine leukemia virus, BLV Env-SU, Deltaretrovirus, Antivirus, Viral interference

## Abstract

Bovine leukemia virus (BLV), a member of the delta retrovirus family, is transmitted horizontally among cows. BLV causes enzootic bovine leukosis and has great economic impact on the cattle industry. Recently, secretory-defective Env proteins (e.g., Refrex-1 and FeLIX) have been detected in domestic cats and shown to possess antiretroviral activity against gammaretroviruses via viral receptor interference. Therefore, we investigated whether BLV-derived molecules engineered similarly exhibit antiviral activity against BLV infection. We generated several proteins consisting of the BLV envelope surface unit (SU) region and signal peptide, without the transmembrane region, and tested their inhibitory effects on BLV infection. These artificial mutant Env-SU proteins were detected as secreted proteins in cultured cells. Colony formation and quantitative PCR assays revealed that the secreted Env-SU proteins exhibited an inhibitory effect on BLV infection. In conclusion, the engineered BLV Env-SU protein was found to effectively inhibit BLV infection, likely through a mechanism consistent with viral receptor interference and is expected to contribute to the development of infection-prevention methods against BLV.

## Highlights

• Bovine leukemia virus (BLV) envelope (Env) proteins without transmembrane region were generated.
• BLV Env-SU (Env-surface unit) proteins inhibited BLV infection.
• Viral interference of BLV was exhibited by the BLV Env-SU protein.

## 1 Introduction

Bovine leukemia virus (BLV) is a deltaretrovirus that causes enzootic bovine leukosis, one of the most prevalent infectious diseases affecting cattle (Sagata et al., 1984). BLV naturally infects cattle and water buffaloes; however, experimental studies have demonstrated its ability to infect various other animal species, including sheep and rabbits (Altanerova et al., 1989; Camargos et al., 2014). The virus is mainly transmitted through infected blood via blood-sucking insects and can also be spread through the sharing of needles and surgical procedures in settings with inadequate decontamination (Gutiérrez et al., 2014). Cow-to-calf transmission occurs via the intrauterine route and milk (Rodríguez et al., 2011). Upon infection, BLV integrates into the host genome as a provirus, establishing a lifelong chronic infection. All BLV infections are exogenous in origin, and no endogenous BLV has been reported (Deschamps et al., 1981).

Most BLV-infected cows are asymptomatic; some have persistent lymphocytosis, whereas <5 % develop neoplastic B-cell lymphoma with apparent clinical signs, including lymphadenopathy, digestive system anomalies, weight loss, and reduced productivity (Hamada et al., 2023; Norby et al., 2016). BLV is distributed worldwide (Lian et al., 2023), with varying prevalence between and within countries (Polat et al., 2017; Rodríguez et al., 2011). Western European countries, such as Denmark, have eradicated the associated disease through combined efforts of testing and culling (Lv et al., 2024). However, the disease still exists in countries, such as the USA, with herd and within-herd prevalences averaging 90 % and 40 %, respectively (Kuczewski et al., 2019). The disease is highly endemic in Japan (Murakami et al., 2013), with >25 % prevalence reported among dairy herds (Murakami et al., 2013) and economic losses resulting from carcass condemnation, reduced milk production, and predisposition to other infections (Nakada et al., 2023).

The BLV genome comprises approximately 8700 nucleotides, including the structural protein-coding genes *gag, pro, pol*, and *env* (Zhao et al., 2025); regulatory genes *tax* and *rex*; and accessory genes *R3*, G4, and miRNA (Aida et al., 2013). The *env* gene encodes two regions: the extracellular region, gp51, referred to as the surface unit (SU), and the transmembrane (TM) region, gp30 (Gillet et al., 2007). Infection is mediated by binding of the SU to the cationic amino acid transporter 1 (CAT1, SLC7A1), similar to the case of the ecotropic murine leukemia virus (eMuLV) (Bai et al., 2019). eMuLV specifically binds to mouse CAT1 and is unable to infect cells expressing human or hamster CAT1 (Kakoki et al., 2014). However, BLV infects cells expressing CAT1 from various animals, including humans, monkeys, and hamsters, indicating that BLV-CAT1 interactions are not species specific (Bai et al., 2019).

Although research on effective prevention strategies for BLV is ongoing, a definitive solution has yet to be established, necessitating the development of alternative therapeutic approaches. In gammaretroviruses, Refrex-1 (Ito et al., 2013) and FeLIX (Pramono et al., 2024) have been shown to effectively suppress viral infection through receptor-mediated interference. Similarly, cells expressing foamy virus Env proteins exhibit resistance to infection by the same virus (Berg et al., 2003). Regarding BLV, superinfection resistance has been confirmed using FLK-BLV and BLV-bat cell lines (Derse and Martarano, 1990). Building on these observations, we investigated the inhibitory effects of truncated secreted BLV envelope proteins on viral infection, aiming to provide fundamental data for the development of novel preventive strategies.

## 2 Materials and methods

### 2.1 Cell culture

The following cells were used in this study: human embryonic kidney transformed with the SV40 large T antigen (HEK293T) (DuBridge et al., 1987), fetal lamb kidney cell line persistently infected with BLV (FLK-BLV) (Deshayes et al., 1977), and HeLa cells (Hirayama et al., 2024). HEK293T and FLK-BLV cells were kindly provided by Dr. Sandra Ruscetti (NCI), and HeLa cells were obtained from Cell Resource Center for Biomedical Research, Institute of Development, Aging and Cancer, Tohoku University. The cells were treated with an anti-mycoplasma agent. The cells were cultured in high-glucose Dulbecco’s modified Eagle’s medium (DMEM; FUJIFILM Wako Pure Chemical Corporation, Osaka, Japan) supplemented with 10 % fetal bovine serum and penicillin–streptomycin (Nacalai Tesque, Inc., Kyoto, Japan).

### 2.2 Construction of BLV Env expression vectors

**Fig. 1:**
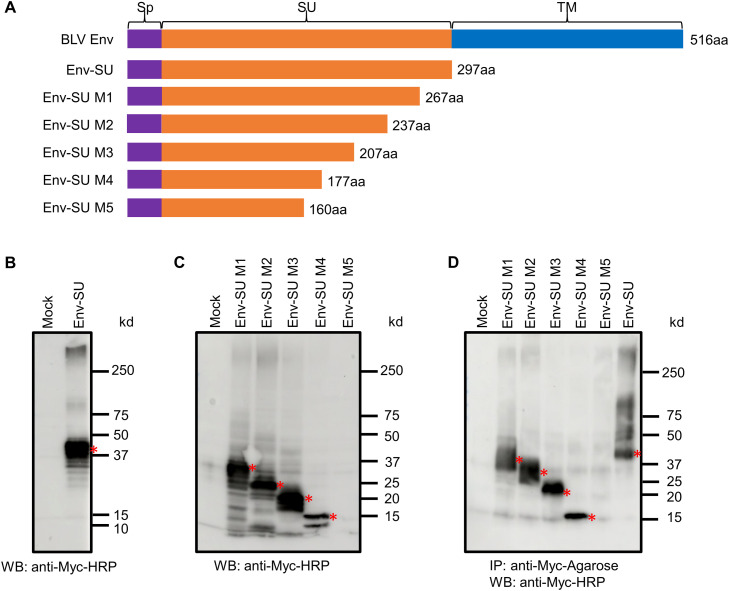
Structure and western blot of Env-SU and deletion mutants. (A) BLV Env-SU (pBLV913; accession EF600696) was codon-optimized to generate Env-SU and mutants M1–M5. Abbreviations: Sp, signal peptide; SU, surface unit; TM, transmembrane; aa, amino acid. HEK293T cells were transfected with empty vector (mock) or Env-SU/M1–M5 constructs. Supernatants were immunoprecipitated with anti-Myc monoclonal antibody covalently conjugated to agarose beads (anti-Myc-Agarose), and western blotting with anti-c-Myc showed protein expression (asterisks) in lysates (B, C) and supernatants (D).

The sequence of BLV Env-SU (pBLV913; accession number EF600696) (Derse, 1988) was genetically synthesized via codon optimization (Eurofins Genomics, Tokyo, Japan) to produce BLV Env SU (Fig. S1). Furthermore, five deletion mutants of BLV Env-SU M1–M5 (Fig. 1A and Fig. S1) were generated using PCR with specific primers (Table 1). All Env-SU proteins were Myc-tagged at the C-terminus. The genes were PCR-amplified using the KOD One ™ PCR master Mix (TOYOBO Bio Inc., Osaka, Japan), digested with *Bam*H1 and *Eco*R1 (Takara Bio, Shiga, Japan), and cloned into the pFU∆ss expression plasmid (DuBridge et al., 1987).

**Table 1:** Primer sequences used for the construction of BLV Env-SUM1–M5 mutants.

**Primer name**	**Primer sequence**	**Primer specificity**
**BLVenvSU F1**	5′-ACCTGAATTCGCCATGCCTAAAAAACGACGGTC-3′	Forward primer
**Fe-959R**	5′-TGCAGAATTCTCACAGATCCTCCTCGGAGATCA GCTTCTGCTCCCTCTGGCTGGGATGATGCC-3′	Env-SU M1
**Fe-960R**	5′-TGCAGAATTCTCACAGATCCTCCTCGGAGATCA GCTTCTGCTCTCCAGATGAGCTGATTGTCT-3′	Env-SU M2
**Fe-961R**	5′-TGCAGAATTCTCACAGATCCTCCTCGGAGATCA GCTTCTGCTCCCTAGCAGTCTGATTCAGGA-3′	Env-SU M3
**Fe-962R**	5′-TGCAGAATTCTCACAGATCCTCCTCGGAGATCA GCTTCTGCTCGGGAATCTTGTGCAGGGAGA-3′	Env-SU M4
**Fe-963R**	5′-TGCAGAATTCTCACAGATCCTCCTCGGAGATCA GCTTCTGCTCCCAGGTCAGGGTGAAAATGC-3′	Env-SU M5

Primers were designed to generate BLV Env-SU deletion mutants (M1–M5) using BLVenvSU F1 as the common forward primer and Fe-959–963R as specific reverse primers.

### 2.3 Preparation of the BLV Env-SU proteins

HEK293T cells were seeded in a six-well plate and transfected with the pFUΔss Env-SU plasmid, its deletion mutants (Env-SU M1–M5), and pFUΔss empty vector using the TransIT®−293 reagent (Takara Bio), according to the manufacturer’s instructions. After 48 h of transfection, cell supernatants were collected, filtered through a 0.22 µm filter (Merck, Darmstadt, Germany), and stored at −80 °C.

### 2.4 Immunoprecipitation and immunoblotting

Cells were lysed in buffer (150 mM NaCl, 20 mM Tris–HCl pH 7.5, 2 mM EDTA, 10 % glycerol, 1 % Triton X-100, 1 mM Na₃VO₄, 1 µg/mL aprotinin, 1 µg/mL leupeptin), incubated on ice (20 min), and centrifuged (4 °C, 12,470 g, 20 min). Supernatants were stored at −80 °C. Protein concentration was measured (Bio-Rad, USA). For immunoprecipitation, supernatants were incubated overnight at 4 °C with anti-c-Myc agarose beads (Clontech, USA). Samples were mixed with reducing buffer (6 ×, Nacalai Tesque), heated (100 °C, 5 min), and resolved by SDS-PAGE (10 %–20 % gels, Invitrogen, Carlsbad, CA 100 V, 2 h). Western blotting was performed using HRP-conjugated anti-c-Myc antibody (FUJIFILM, Japan) and LumiGLO® substrate (Cell Signaling Technology). Blots were imaged using LAS2000 (Fujifilm) or ImageQuant 800 (Cytiva, Shinjuku, Japan).

### 2.5 BLV preparation

FLK-BLV cells stably transfected with pBLV-SVNEO (Derse and Martarano, 1990), which contained the neoresistance gene, were used as a BLV source, as previously described (Derse and Martarano, 1990). The pBLV-SVNEO was kindly provided by Dr. David Derse (NCI). The cell supernatants were collected, filtered through a 0.22 µm filter (Merck), and stored at −80 °C.

### 2.6 Colony formation assay of BLV in the presence of Env-SU protein

HeLa cells were seeded in 12-well plates, treated with 450 µL supernatant for 2 h, then inoculated with 180 µL BLV plus 10 µg/mL polybrene (Nacalai Tesque, Inc.) for 48 h. Cells were transferred to six-well plates and cultured with 600 µg/mL G418 for ∼14 days before resistant colonies were counted.

### 2.7 Quantitative PCR for BLV

HeLa cells in 24-well plates were treated with 250 µL BLV Env-SU, deletion mutants, or mock supernatant for 2 h, then infected with 250 µL BLV for 48 h. Cell counts were obtained using the EVE™ Automated Cell Counter (NanoEnTek, Korea). DNA was extracted with the DNeasy® Blood and Tissue Kit, and BLV quantified by qPCR (Takara Bio Detection Kit). PCR conditions were 25 °C for 10 min (UDG activation), 95 °C for 30 s (denaturation), and 45 cycles of 95 °C for 5 s and 60 °C for 30 s. Reactions were run in triplicate on a CFX96 Touch Real-Time System (Bio Rad), with standard curves from 10-fold plasmid dilutions.

### 2.8 Statistical analysis

Data are presented as mean with standard deviation in all bar diagrams. Statistical significance of the assay results was determined using Welch’s *t*-test, with *p* < 0.05 considered significant.

### 2.9 Ethical approval

All the experiments were approved by the Genetic Modification Safety Committee of Yamaguchi University.

## 3 Results

### 3.1 BLV exhibits superinfection resistance

FLK-BLV cells were infected with BLV, followed by neomycin selection. After 12 days of antibiotic selection, no colonies or surviving cells were observed. This observation is consistent with the findings of a previous report (Derse and Martarano, 1990), suggesting that BLV-infected cells exhibit a degree of resistance to superinfection.

### 3.2 Expression of BLV Env-SU protein in HEK293T cells

We aimed to purify the BLV Env-SU protein, comprising the envelope SU and signal peptide, which includes the receptor-binding domain of the virus. The protein spans 297 amino acids (891 nucleotides; Fig. S1, S2) and is expected to be secreted. An initial expression vector using a non-optimized BLV sequence failed to yield protein expression. We therefore synthesized a codon-optimized gene with a Myc tag at the 3ʹ-end and transfected it into HEK293T cells. Immunoprecipitation and western blotting with anti-Myc antibody detected a ∼40 kDa Env-SU protein in both cell lysate and supernatant (Fig. 1B, 1C). Additional bands at 50 and 70 kDa were observed in the supernatant, although their identities remain unclear. These results confirm that codon optimization enabled expression and secretion of wild-type BLV Env-SU.

### 3.3 BLV Env-SU protein inhibits BLV infection

After confirming Env-SU expression in HEK293T cells, we investigated its inhibitory effects against BLV infection. HeLa cells are highly susceptible to BLV infection (Derse and Martarano, 1990). Therefore, HeLa cells pretreated with Env-SU protein from the supernatant of 293T cells transfected with the Env-SU expression plasmid were infected with BLV containing the neomycin-resistant marker gene (*BLV-SVNEO*). Cells were cultured in the presence of the G418 antibiotic for 14 days, and G418-resistant colonies were counted. The number of colonies in the test and control groups were 0–4 and 58.5–79, respectively (Table 2). The colony formation assay results indicated that BLV Env-SU significantly inhibited BLV infection (*p* < 0.001).

**Table 2:** Inhibitory effects of Env-SU on BLV infection determined by colony-forming assays.

**Env protein**	**Number of G418-resistant colonies**				**Statistical analysis**
	1st time	2nd time	3rd time	4th time	
**Mock**	58.5 ± 29	73.3 ± 24.2	79 ± 7	78 ± 2.8	
**Env-SU**	0	0.7 ± 1.2	2.3 ± 1.5	4 ± 2.8	*p* < 0.001

Values represent the mean ± SD of four independent experiments. *p*-values were calculated using Welch’s *t*-test, comparing each Env-SU group to the Mock control.

Subsequently, we investigated the relationship between the concentration of BLV Env-SU and its ability to inhibit BLV infections. The amount of BLV Env-SU was diluted, and the inhibitory effect was examined. A significant reduction in the number of G418-resistant colonies was observed at a 10-fold dilution (*p* < 0.05) (Table 3), indicating that BLV Env-SU exhibits a dose-dependent inhibitory effect against BLV infection.

**Table 3:** Dose-dependent inhibitory effects of Env-SU on BLV infection determined by colony-forming assays.

Env protein	**Number of G418-resistant colonies**	**SD**	**Statistical analysis**
**Mock**	99.7	12.5	
**Env-SU 4.5 µL**	95.3	9.5	0.78
**Env-SU 45 µL**	39.3	2.1	<0.01
**Env-SU 90 µL**	17.6	3.9	<0.01
**Env-SU 225 µL**	12.3	2.6	<0.01
**Env-SU 450 µL**	13.7	6.6	<0.01

The Env-SU protein was added to the virus infection inhibition assays in volumes ranging from 4.5 µL to 450 µL, as indicated in the table.

Values represent mean ± SD (*n* = 3). *p*-values were calculated using Welch’s *t*-test, comparing each Env-SU group to the Mock control.

Next, five BLV Env-SU mutants with C-terminus deletions were constructed from the Env-SU based on BLV Env-SU. Env-SU M1–5 consisted of 267, 237, 207, 177, and 160 amino acid residues, respectively (Fig. 1A, and Fig. S1). Experiments using these mutants determined whether the length of Env-SU influenced its inhibitory effect on BLV infection. All mutant proteins were expressed in cell lysates and as soluble proteins in the supernatants of HEK293T cells, except for Env-SU M5 (Fig. 1C and D). Four mutant proteins (M1–M4) were detected in the culture supernatant as single bands of approximately 37, 25, 20, and 15 kDa, respectively. In the cell lysates, the Env-SU mutant proteins were strongly detected as a broad band, accompanied by minor bands. The effects of the four BLV SU mutants (Env-SU M1–M4) on BLV infection were determined using the colony formation assay. After 14 days of cell culture under G418 antibiotic selection, the average number of colonies in the test and control groups was 0 and 48.3 ± 4.9, respectively (Table 4). This significant difference (*p* < 0.001) indicated that the BLV Env-SU mutants effectively protected target cells against BLV infection. The Env-SU M4 mutant protein, consisting of 177 amino acids, effectively inhibited viral infection as the smallest protein.

**Table 4:** Inhibitory effects of Env-SU mutants (M1–M4) on BLV infection determined by colony-forming assay.

**Env protein**	**Mean**	**SD**	**n**	**Statistical analysis**
**Mock**	48.3	4.9	3	
**Env-SU M1**	0	0	3	*p* < 0.001
**Env-SU M2**	0	0	3	*p* < 0.001
**Env-SU M3**	0	0	3	*p* < 0.001
**Env-SU M4**	0	0	3	*p* < 0.001

Values represent the mean ± SD of three independent replicates. *p*-values were calculated using Welch’s *t*-test, comparing each Env-SU mutant group to the Mock control.

Next, to verify the inhibitory effect of the BLV Env-SU proteins using an alternative method, we quantitatively measured the number of BLV proviral copies during viral infection using PCR. Our results revealed that the mean BLV copy number (normalized per 10^6^ live cells) was 358.2 ± 46.9 in the control group but this was markedly reduced to 19.4 ± 2.5 in the presence of wild-type Env-SU. Similarly, the BLV copy numbers in the presence of the Env-SU mutants M1–M4 were 14.3 ± 17, 10.3 ± 14.4, 31.5 ± 11.5, and 13.0 ± 6.1, respectively (Table 5), showing a significant difference (*p* < 0.01) in infection levels compared to the mock. These findings suggest that Env-SU and its truncated mutants (minimum length of 177 amino acids) effectively suppress BLV infection.

**Table 5:** Inhibitory effects of Env-SU mutants (M1–M4) on BLV infection determined by quantitative PCR.

Env protein	**Mean BLV copy number/10^6^ cells**	**SD**	**Statistical analysis**
Mock	358.2	46.9	
Env-SU	19.4	2.5	*p* < 0.01
Env-SU M1	14.3	17	*p* < 0.01
Env-SU M2	10.3	14.7	*p* < 0.01
Env-SU M3	31.5	11.5	*p* < 0.01
Env-SU M4	13	6.1	*p* < 0.01

Values represent the mean ± SD of three independent replicates. *p*-values were calculated using Welch’s *t*-test, comparing each Env-SU mutant group to the Mock control (*n* = 3 per group).

## 4 Discussion

BLV is globally prevalent, including in Japan, causing major economic losses and requiring urgent countermeasures. No vaccine or effective antivirals exist. We assessed BLV envelope proteins for inhibitory potential, informed by evidence in humans, cats, and mice where defective envelope proteins act via receptor-mediated mechanisms (Kassiotis and Stoye, 2025; Miyake et al., 2022). Viral interference is well established in alpharetrovirus, gammaretrovirus, and spumaretrovirus (Nethe et al., 2005) but less defined in deltaretroviruses; BLV, a deltaretrovirus, shows interference (Maezawa et al., 2024), which we confirmed by colony formation assays. Codon optimization enhanced protein expression, and truncated BLV envelope proteins demonstrated protective activity against infection.

### 4.1 Protein expression of BLV envelope proteins

We initially failed to express wild-type Env-SU and defective mutants (M1–M4) in HEK293T cells. Using artificial gene synthesis and codon optimization, we achieved successful expression of Env-SU and generated the four mutants (M1–M4) from the codon-optimized construct.

Codon optimization is a key factor in protein expression. While rare codons—such as those for arginine, glycine, cysteine, and isoleucine—can limit yield by causing translation errors or premature termination (Sørensen and Mortensen, 2005), they also play a functional role in regulating translation speed. Specifically, programmed ribosome stalling at rare codons can facilitate the proper folding of nascent polypeptides. Given that not all proteins fold within the endoplasmic reticulum, this translation-coupled folding is essential for maintaining structural integrity. Thus, while we employed codon optimization to enhance productivity (Gao et al., 2022; Tomé-Poderti et al., 2024), we remained mindful of its complex effects on protein folding and stability.

BLV Env-SU (297 amino acids) and the mutant proteins (177–267 amino acids) were detected within cells and expressed as soluble proteins in the culture supernatants. Notably, BLV Env-SU proteins purified from cell culture supernatants were detected as single bands. Although Env-SU was purified as the intended protein, the presence of bands at 50 kDa and 70 kDa suggests the possibility of dimer formation. The BLV receptor-binding domain is estimated to consist of 176 amino acids (Gatot et al., 2002) (Fig. S2), and all variants expressed in HEK293T cells contained the receptor-binding domain, except the fifth variant (160 amino acids). The Env-SU M5 mutant remained undetectable in both cell lysates and supernatants. As our western blot conditions reliably resolved proteins of this size (∼15 kDa), this absence is unlikely to be a technical artefact. Instead, the complete loss of the receptor-binding domain likely causes severe misfolding (Gao et al., 2022). Such structural instability typically triggers rapid intracellular degradation via quality-control pathways, preventing the protein from accumulating to detectable levels.

### 4.2 Inhibitory effect of BLV Env-SU

Similar to other retroviruses, the BLV *env* gene encodes two functional proteins: SU subunit and TM subunit. The first step of virus infection involves the interaction of the SU protein with cell surface receptors (Overbaugh et al., 2001). Regardless of whether infection occurs via cell-free viruses or cell-to-cell transmission, BLV Env-SU interacts with CAT1 on the cell surface (Bai et al., 2019; Sato et al., 2020). In the present study, the codon-optimized BLV Env-SU and four of its deletion mutants successfully inhibited BLV infection in HeLa cells. Although a direct interaction between Env-SU and its receptor (CAT1) was not explicitly demonstrated, this inhibition could potentially result from competitive binding to the target cell receptor, thereby blocking viral entry.

Refrex-1 is a truncated soluble envelope protein encoded by the *env* genes of the endogenous feline gammaretrovirus (ERV-DC) in domestic cats. Refrex-1 demonstrated protective effects against both endogenous and exogenous retroviruses (Ito et al., 2013). Using a structural model of Refrex-1 generated as a soluble protein, we artificially created Env-SU proteins. Env-SU contains a signal peptide and the N-terminal region of Env; however, it does not possess the TM region. As a result, this protein exhibits intracellular expression and extracellular secretion.

However, the exact mechanism by which Env-SU suppresses BLV infection remains unclear. We hypothesized that soluble Env-SU protein binds to the BLV viral receptor CAT1 on the cell membrane and inhibits viral infection through a receptor-binding competition mechanism. CAT1 is a highly hydrophilic protein consisting of 622 amino acids and is widely expressed (Bai et al., 2019). It functions as a transporter for essential amino acids, including histidine, arginine, lysine, and ornithine (Fotiadis et al., 2013; Okita et al., 2021). Considering the effects of Env-SU protein on CAT1, investigating its effects in animal models may provide valuable insights.

BLV infects cells via cell-free and cell-to-cell infection (Jahan et al., 2025; Watanuki et al., 2019). This study examined only cell-free infection; quantification of cell-to-cell spread via syncytium formation was unsuccessful, requiring further investigation. Several bovine molecules show antiviral activity against BLV and other retroviruses. Interferon-tau reduces BLV syncytium formation with minimal cytotoxicity (Kohara and Yokomizo, 2007); bBST-2 isoforms inhibit BLV and vesicular stomatitis virus (Takeda et al., 2012); and TIM-3/PD-1 blockade enhances IFN-γ production and antiviral efficacy (Nakamura et al., 2023). Combining these molecules with optimized Env-SU—either as standalone treatments or to exploit potential synergistic effects—may significantly aid future BLV prevention strategies.

In this study, viral quantification was performed using the colony formation and PCR methods for cell-free viruses (Derse and Martarano, 1990; Pluta et al., 2024). Although the colony formation method is time-consuming compared to PCR, it is substantially more cost-effective for viral quantification. Although at least 11 BLV genotypes (G1–G11) are documented (Yu et al., 2019), whether genotype variation alters viral interference or Env-SU inhibition remains unknown, highlighting the need for further study. Furthermore, the therapeutic potential and cytotoxicity reported herein need to be evaluated in vivo. Purification of our mutants and administration to animal models that should be subjected to BLV infection thereafter should be carried out to determine the efficacy of these mutants. Although representing idealized strategies, our findings demonstrate that engineered Env-SU proteins could serve as a versatile foundation for future BLV control. Specifically, these constructs hold potential for passive immunization to block viral entry, can serve as potent vaccine antigens to elicit robust neutralizing antibodies, or can be used in genetic engineering to confer sustained resistance through constitutive expression. These applications underscore the potential of our optimized Env-SU to contribute significantly to effective measures for preventing BLV transmission.

In conclusion, BLV Env-SU expression reduced BLV infection in vitro, although the precise mechanism—whether receptor competition or another pathway—remains to be determined.

## Funding

This study was funded by the 10.13039/501100001691Japan Society for the Promotion of Science, KAKENHI (grant numbers 23K27086 and 23H02393 to KN).

## CRediT authorship contribution statement

**Nashon Wanjala:** Writing – review & editing, Writing – original draft, Validation, Investigation, Formal analysis. **Ryusuke Matsumoto:** Investigation, Formal analysis. **Didik Pramono:** Validation, Methodology, Investigation, Data curation. **Ariko Miyake:** Investigation. **Kazuo Nishigaki:** Writing – review & editing, Writing – original draft, Visualization, Validation, Supervision, Project administration, Methodology, Investigation, Funding acquisition, Formal analysis, Data curation, Conceptualization.

## Declaration of competing interest

The authors declare no conflicts of interest.

## Data Availability

Data will be made available on request.

## Appendix Supplementary materials

Image, application 1
