# Supplementary material for: Engineered soluble truncated envelope proteins block bovine leukemia virus infection
Source: Virus Res. 2026 Feb 5;365:199701. doi: 10.1016/j.virusres.2026.199701 (PMC12908037; doi:10.1016/j.virusres.2026.199701)
Supplement: Supplementary file 1 [file mmc1.docx]

**Supplementary Materials**

**
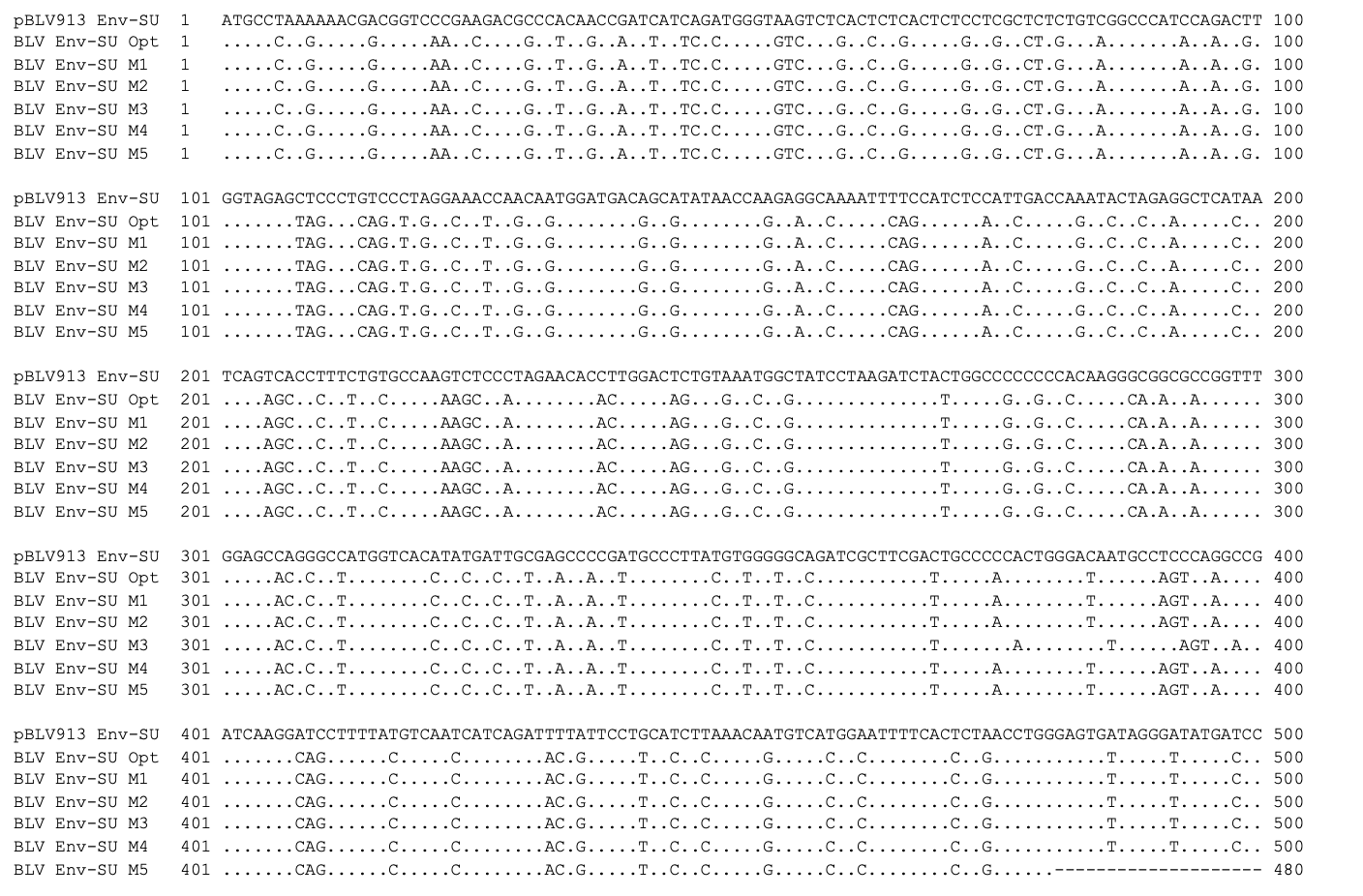
**

**
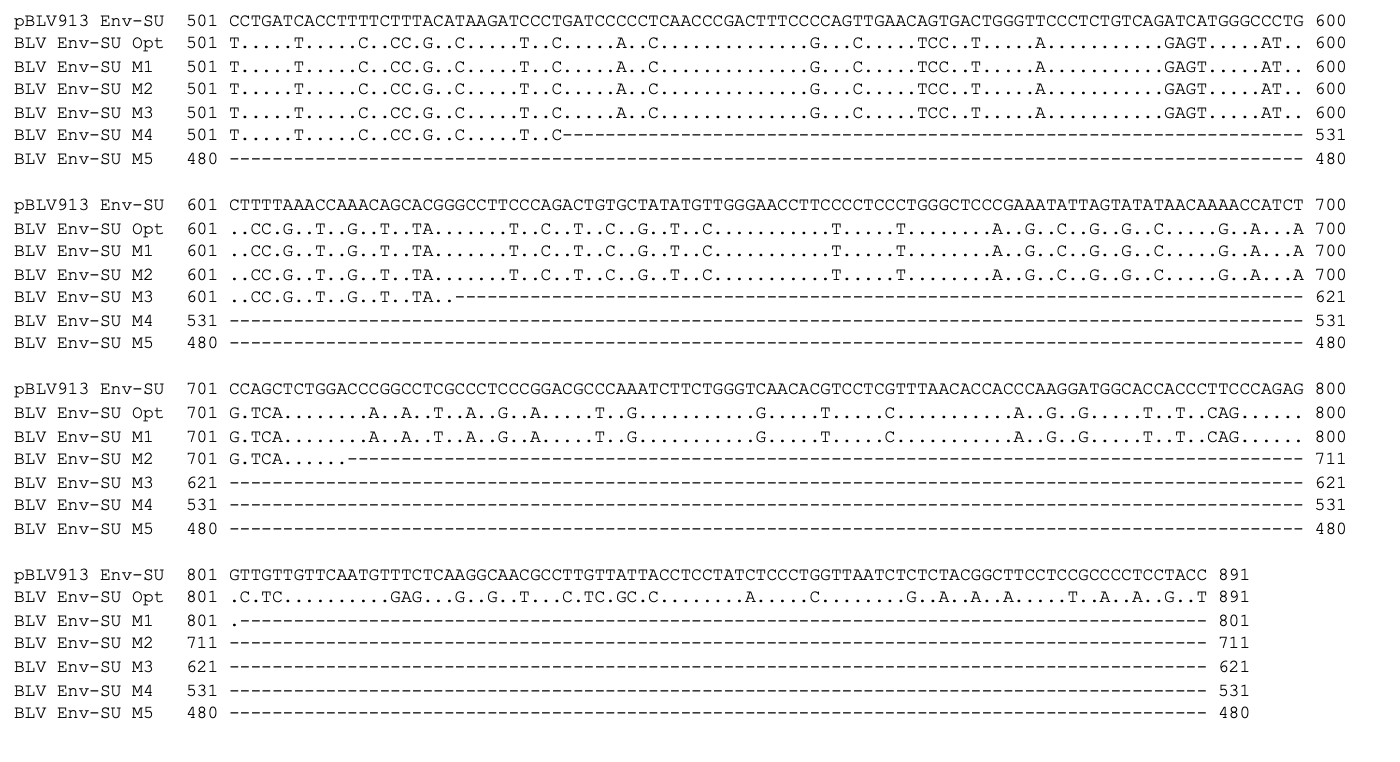
**

**Figure S1**. Nucleotide sequence alignment of BLV Env-SU and its mutants. The sequence of the plasmid pBLV913 (GenBank accession number EF600696) was used to construct BLV Env-SU, which was codon-optimized by gene synthesis to produce the optimized BLV SU used in this experiment. The optimized BLV Env-SU was used to construct five deletion mutants BLV Env-SU M1–M5 (801, 711, 621, 531, and 480 nucleotides, respectively). BLV, bovine leukemia virus; SU, surface unit.


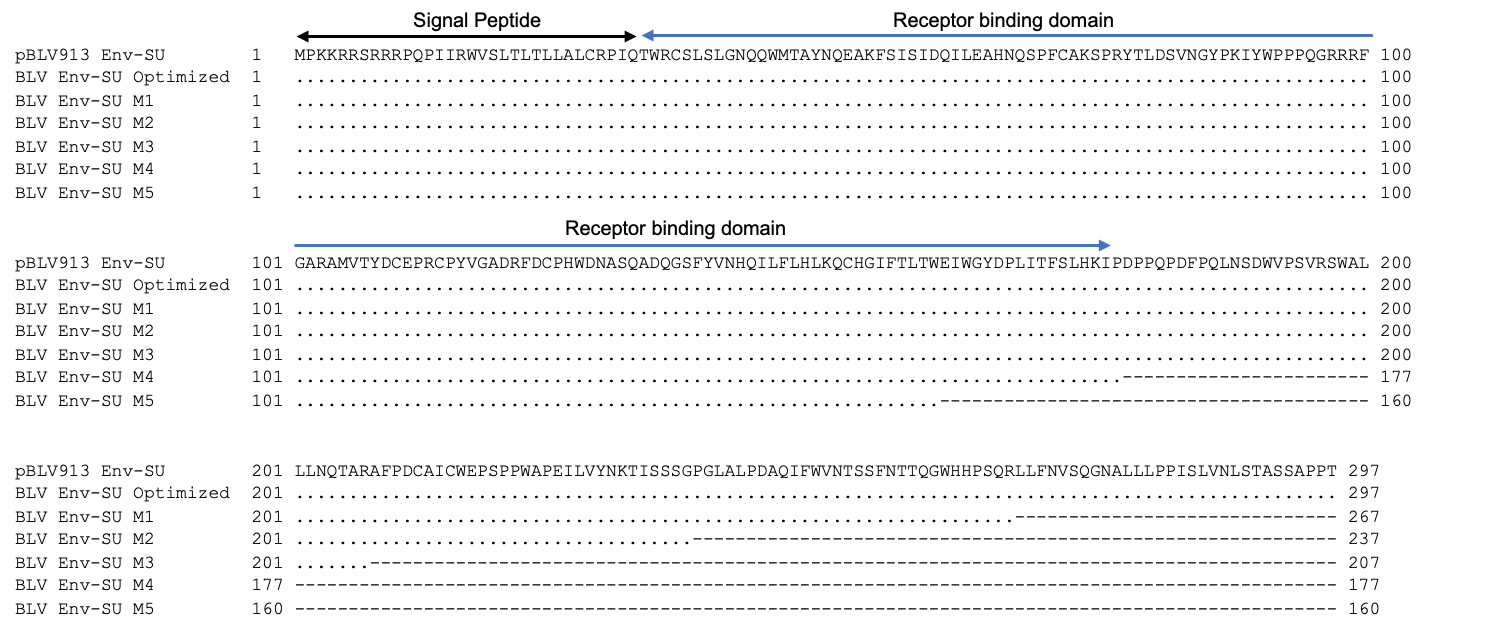


**Figure S2**. Alignment of the amino acid sequences of BLV Env-SU and its mutants. The sequence of the plasmid pBLV913 (GenBank accession number EF600696) was used to construct BLV Env-SU, which was codon-optimized by gene synthesis to produce the optimized BLV Env-SU used in this experiment. The optimized BLV Env-SU was used to construct five deletion mutants BLV Env-SU M1–M5 (267, 237, 207, 177, and 160 amino acids, respectively). The text shows the viral receptor-binding domain, including the signal peptide. BLV, bovine leukemia virus; SU, surface unit
